# Treacher-Collins Syndrome With Anorectal Malformation: A Rarity and a Challenge for the Surgical Team

**DOI:** 10.7759/cureus.100001

**Published:** 2025-12-24

**Authors:** Mary-ann O Monyei, Morrison E Edena, Felix Olodiama, Obi A Ekene

**Affiliations:** 1 Surgery, University of Benin Teaching Hospital, Benin City, NGA

**Keywords:** anaesthetic chanllenge, anorectal malformation, congenital, southern nigeria, treacher-collins syndrome

## Abstract

Treacher-Collins syndrome (TCS) is a rare anomaly involving the first and second pharyngeal arches, resulting in structural anomalies to the bones and soft tissue in the region of the face, including the ears and pharynx. Due to the peculiarities of the face and pharynx, this condition presents an interesting challenge to the pediatric anesthetist. Anorectal malformation (ARM) is one of the most common causes of neonatal intestinal obstruction requiring emergency surgery. To our knowledge, there is no known association between TCS and ARM, and no reports of both conditions occurring in the same patient.

We present a case of TCS in a child with ARM, highlighting the challenges of surgical and anesthetic management in such neonates, particularly the need for securing the airway in a low-resource setting. A 2.3-kg, three-hour-old neonate with distinctive facial anomalies, including poorly developed external ears, telecanthus, a flat nasal bridge, a high-arched palate, and an absent anal opening, was diagnosed with a high ARM without fistula. A decision was made to pursue staged management of the ARM, with initial colostomy creation. However, surgery was delayed until the sixth day due to repeated failed attempts at intubation. This necessitated the use of general anesthesia delivered via facemask, given the limitations of the setting.

This case highlights the challenges posed by mandibulofacial anomalies causing difficult intubation, as seen in TCS, which are further compounded by the need for emergency surgery in neonates. Additionally, we draw attention to the rare occurrence of ARM in a child with TCS.

## Introduction

In 1900, Treacher described a series of anomalies in two children who presented with characteristic ocular and periorbital deformities [1]. Since this initial description, several studies have documented this rare anomaly, also known as Treacher-Collins-Franceschetti syndrome or mandibulofacial dysostosis. It is a rare autosomal dominant congenital malformation involving the first and second pharyngeal arches, resulting in multiple craniofacial deformities affecting the bones and soft tissues of the face, ears, mouth, and airway [1,2]. It has a global incidence of 1:40,000-70,000 live births [3].

Its etiology has been mapped to an abnormality affecting chromosome 5q (5q31.3-q33.3) [4]. These genes code for proteins that are involved in the ribosome biogenesis pathway; hence, mutations result in abnormal development of the facial bones and other structures of the face [4]. It is not yet clear why the abnormality appears to be limited to the face. The remaining cases have an as-yet-unidentified cause of the condition. Phenotypic expression varies from almost unnoticeable to severe. It is characterized by bilateral abnormalities, including a triad of micrognathia, glossoptosis, and posterior cleft palate, and deformed external ears may be identified on prenatal ultrasound. Postnatal examination may reveal a hypoplastic mandible (microretrognathia), dysmorphic orbital bones, down-slanting palpebral fissures, malar hypoplasia, malformed ears with low-set auricular remnants, stenosis or atresia of the external auditory meatus, and middle and inner ear deformities [3]. A combination of microretrognathia, a small oral cavity, a cleft or high-arched palate, and hypoplastic pharyngeal and laryngeal structures contributes to difficulty with both mask ventilation and intubation in these patients. They are therefore likely to require advanced airway tools, such as fiberoptic laryngoscopes or bougies (tracheal introducers), when intubation is necessary. Such tools, in sizes appropriate for neonates, are less likely to be available in low-resource settings.

Anorectal malformations are best described as a spectrum of congenital anomalies involving the anorectum, with or without involvement of the genitourinary system and sometimes associated with sacral spine and cord abnormalities, manifesting as absence of a normal anal opening and resulting in intestinal obstruction. These malformations may be described as *high* when the lowest pouch of rectal gas is seen on an appropriate X-ray (cross-table lateral in the jack-knife position, performed at least 24 hours after birth) more than 1 centimeter above the perineal skin. This indicates that such a child would require staged management, with creation of an initial colostomy during the neonatal period, followed by definitive surgery (posterior sagittal anorectoplasty) later in life. We report an atypical association of Treacher-Collins syndrome (TCS) with high anorectal malformation to highlight the challenges of managing a neonate with mandibulofacial dysostosis and an emergency surgical condition, particularly in a low-resource setting.

## Case presentation

**Figure 1:**
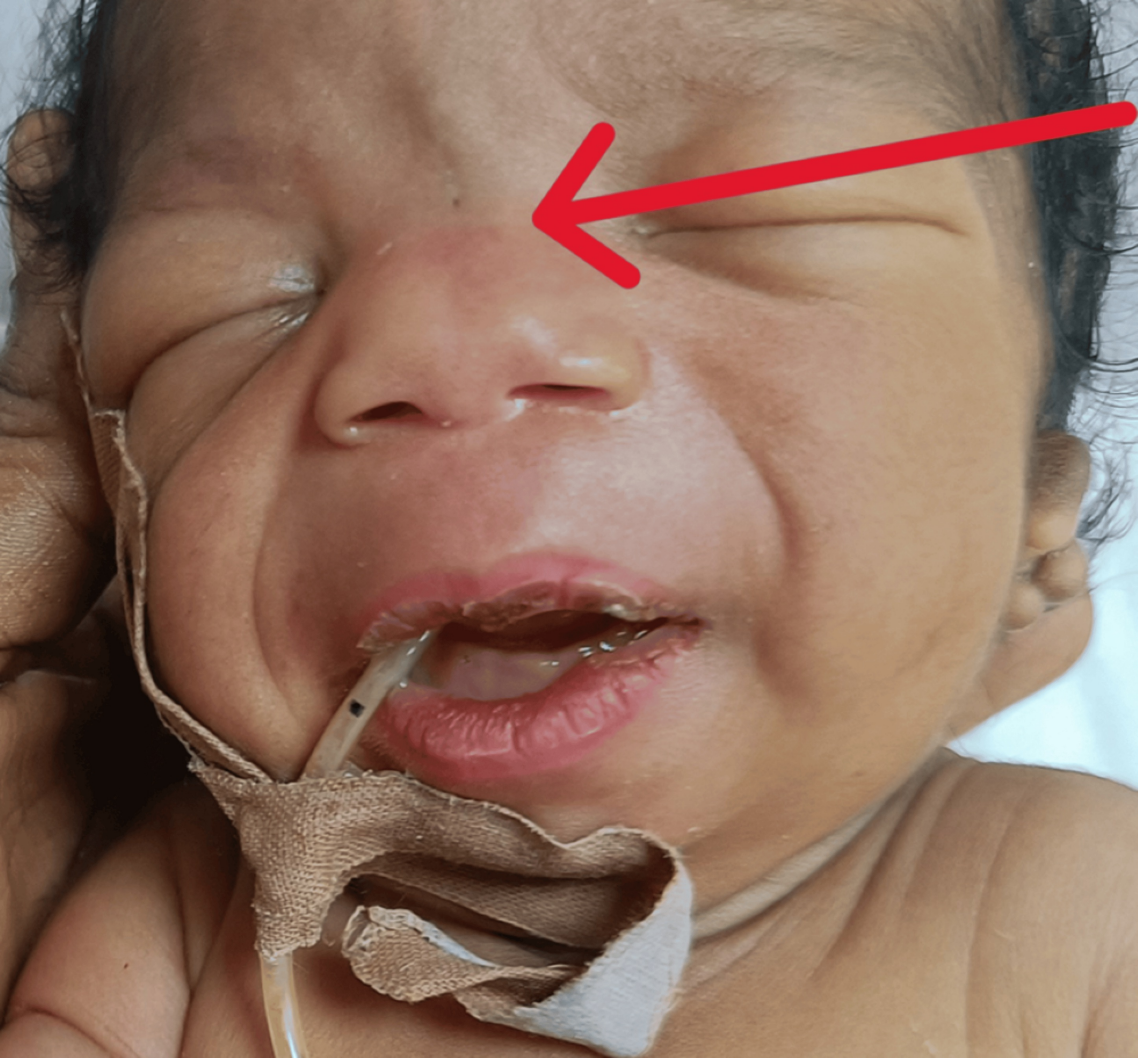
Anterior view of the neonate. The red arrow indicates telecanthus and a flattened nasal bridge. Note the down-slanting palpebral fissures.

**Figure 2:**
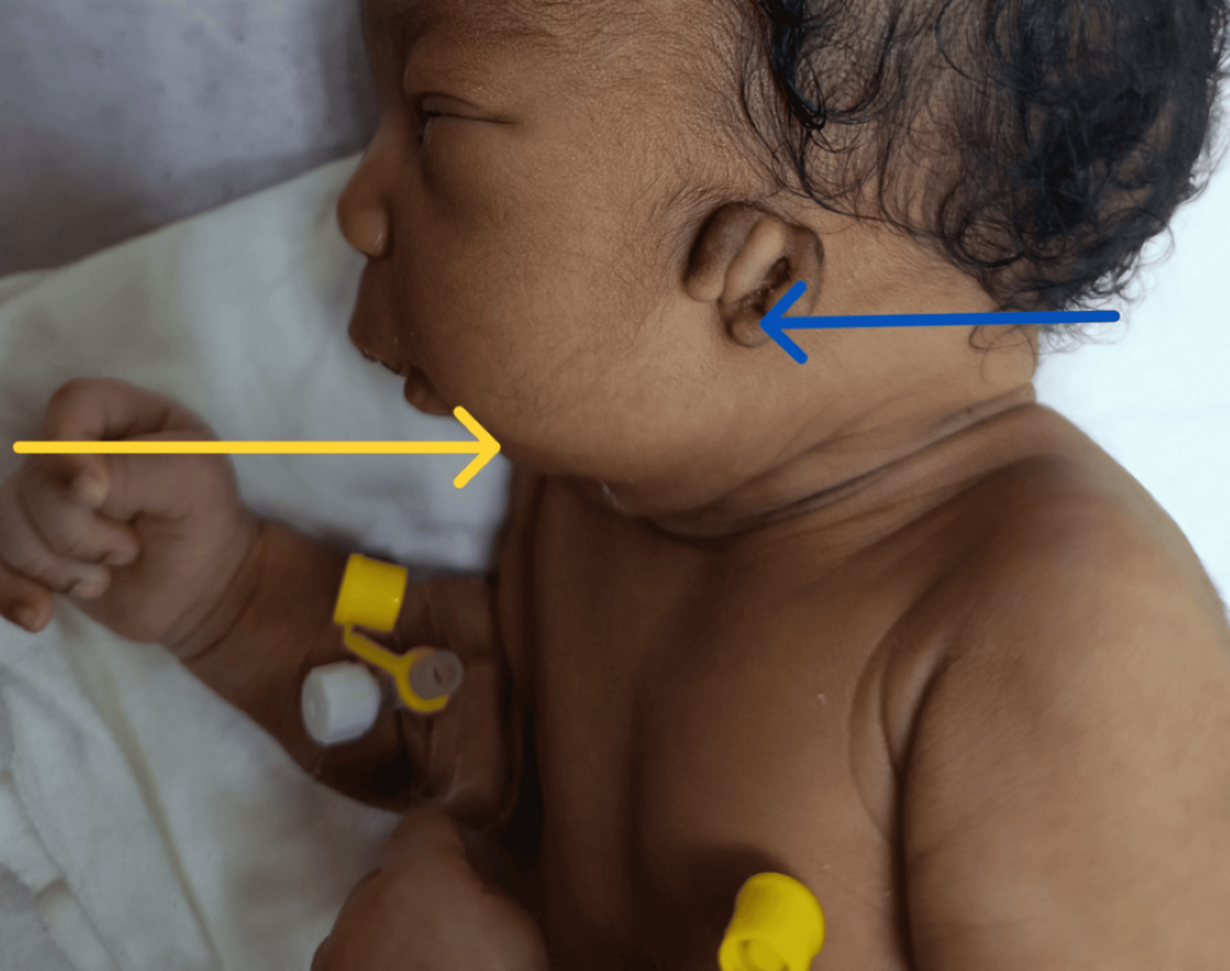
Lateral view of the newborn showing features consistent with Treacher-Collins syndrome. Blue arrow: low-set, underdeveloped auricular remnants present bilaterally. Yellow arrow: microretrognathia.

**Figure 3:**
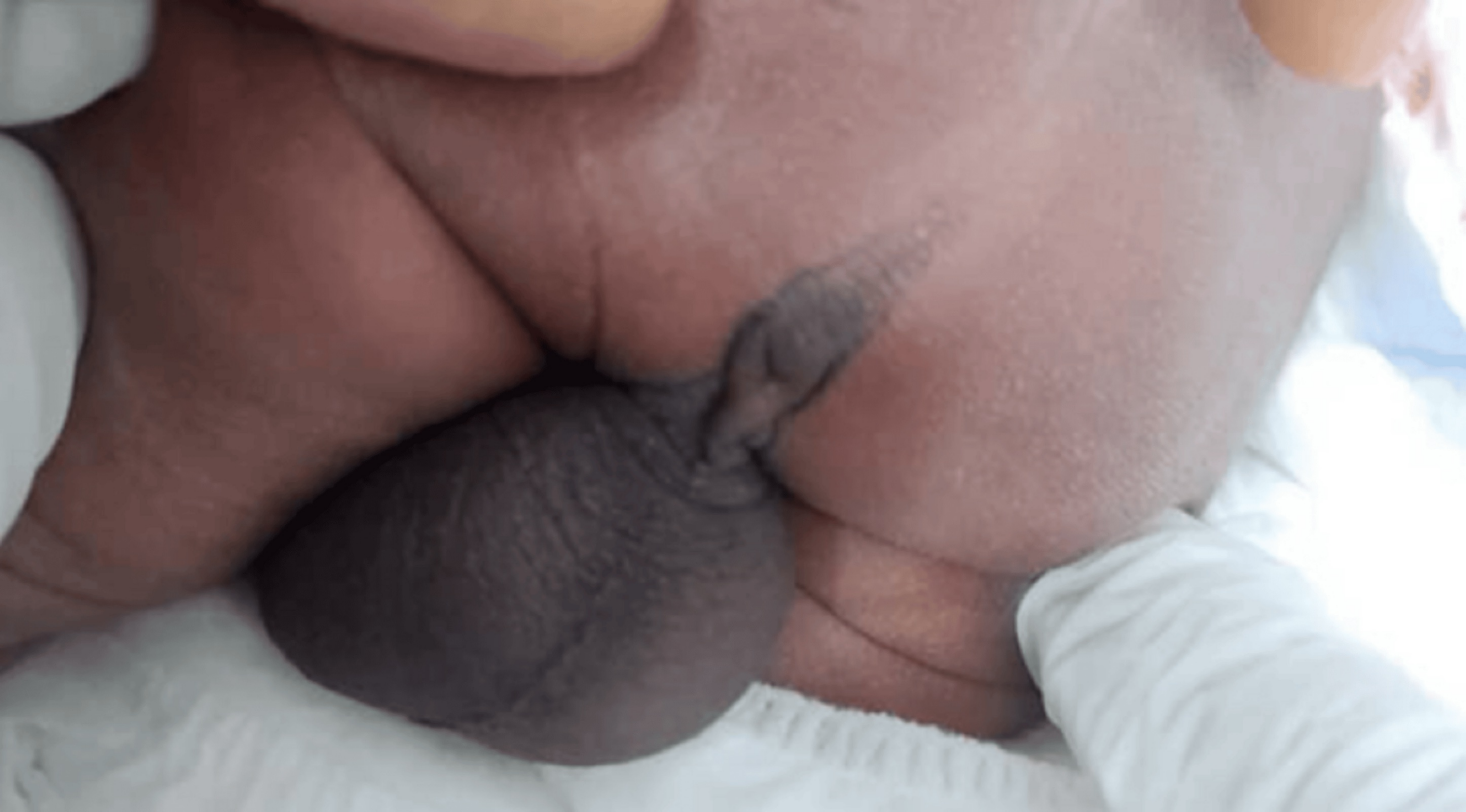
Absent anal opening in a male neonate. Flattened buttocks and poorly developed gluteal folds, suggestive of a high anorectal malformation.

A 2.3-kg male neonate was seen 3 hours after birth. He had been delivered via elective cesarean section at 38 weeks. The APGAR score was 8 at 1 minute and 9 at 5 minutes. A single prenatal ultrasound performed at 16 weeks showed no fetal abnormalities or abnormal amniotic fluid volume. The mother had taken alcohol-based herbal preparations and antiretroviral drugs (tenofovir-lamivudine-efavirenz), in addition to her routine hematinics. She had experienced three previous pregnancy losses at 8, 11, and 26 weeks; however, the cause had never been investigated. Notably, the mother had a mild facial anomaly, with telecanthus and a flat nasal bridge. Postnatal examination of the neonate revealed features consistent with TCS, including microretrognathia, glossoptosis, cleft palate, down-slanting palpebral fissures with hypertelorism, and low-set auricular remnants (Figures 1-2). Examination of the perineum showed the absence of the anal opening, with poorly developed gluteal muscles and folds (Figure 3).

Echocardiogram revealed a structurally normal heart. Abdominal ultrasound revealed bilateral unexplained moderate hydroureteronephrosis. Gauze left around the penis demonstrated the absence of meconuria (no evidence of urinary fistula). A cross-table lateral X-ray in prone jackknife position was done at 26 hours of life and revealed a high anorectal malformation. This meant that a primary corrective surgery could not be done; instead, the parents were counselled as appropriate.

A decision was made for staged management of the conditions, beginning with creation of an initial sigmoid Devine colostomy to decompress the bowel. From the third to fifth day of life, repeated attempts at intubation were made. Although the airway could not be secured due to repeated failed intubation, which we attributed to the abnormal mandible, small oral cavity, and hypoplastic pharyngeal and laryngeal structures that prevented visualization of the epiglottis and vocal cords using a traditional straight Miller laryngoscope, the child could be ventilated between attempts using a facemask, maintaining oxygen saturation between 97% and 100%. However, the abdomen became progressively distended, causing respiratory compromise due to the unresolved intestinal obstruction. Appropriately sized bougie, video laryngoscope for a neonate, and a fibre-optic bronchoscope were unavailable. Tracheostomy was considered and discussed with an otolaryngologist (ORL) and the family; however, a tracheostomy tube of suitable size for the neonate was not available and would have to be ordered. Additionally, the parents were counseled about the anticipated difficulty of decannulating an infant of that age and the possibility of long-term tracheostomy, particularly if required for subsequent surgeries. Discussions were held among the family, surgical team, anesthesiologists, and neonatologists regarding the limited anesthesia options available. The child eventually underwent surgery on the sixth day of life under general anesthesia (halothane) delivered via facemask, with otorhinolaryngologists on standby for tracheostomy in case of difficulty ventilating. Recovery from anesthesia was uneventful; oxygen saturation on room air remained between 98% and 100% with good bilateral breath sounds, suggesting a low risk of aspiration.

The colostomy became functional 12 hours later. The child was discharged home on the tenth day of life, with a plan for posterior sagittal anorectoplasty at six months of age. However, the child presented three weeks after colostomy creation with a non-functioning stoma and progressive abdominal distention. An attempt to wash out the proximal limb of the stoma failed to relieve the symptoms. Abdominal X-rays showed multiple air-fluid levels with thickened small bowel walls, suggesting adhesive small bowel obstruction. Resuscitative measures were initiated, with a plan for exploratory laparotomy. Unfortunately, the child suffered a cardiorespiratory arrest.

## Discussion

The numerous developmental anomalies described in relation to TCS mostly affect the head and neck (first and second pharyngeal arch structures) [5]. Other conditions presenting with similar facial anomalies and considered close differentials include Nager syndrome, which features absent or hypoplastic thumbs and radial-ulnar fusion; Miller syndrome, which, in addition to facial anomalies, presents with lower lid ectropion and limb defects; Goldenhar syndrome, characterized by notable facial asymmetry; and Pierre-Robin sequence, a *chain reaction* of anomalies resulting from oligohydramnios. The majority (over 80%) of cases affect the TCOF1 gene, and POLR1C and POLR1D gene abnormalities are seen in approximately 2% of cases. These genes code for a protein (Treacle), which is critical for ribosomal RNA synthesis. The reason for the limitation of these anomalies to the face is unknown. These deformities create a small oral cavity, which, in addition to a retrognathic mandible, posterior-superior tongue displacement, and a high-arched palate, makes for a difficult airway.

A combination of the above creates a difficult view of the glottis, corresponding to Cormack-Lehane class III or IV, where the experienced physician sees only the epiglottis or neither the epiglottis nor the glottis, respectively [6]. This makes intubation and ventilation challenging. Additional malformations described in children with TCS include absence of the parotid gland, abnormalities of the cervical spine, malformed extremities, cryptorchidism, renal anomalies, and congenital heart disease [7].

In our extensive search, we found a few reported cases of TCS from Africa [8-9]. All of these were diagnosed on the basis of strong clinical suspicion due to the absence of facilities for extensive genetic testing. There were no previously published records of anorectal malformation in a patient with TCS or other mandibulofacial anomalies [5]. The maternal use of the antiviral drug combination Tenofovir-Lamivudine-Efavirenz, as well as multiple (three) early pregnancy losses while on the same regimen, may be a significant etiology of this rarity and, as such, warrants further research.

The occurrence of a neonatal surgical emergency, exemplified by anorectal malformation, in association with mandibulofacial dysostosis in the index patient created a management dilemma. Many children with TCS (up to 41%) will eventually require tracheostomy, but only about 12%-18% of these will need it in the neonatal period [10]. However, this added need for airway establishment and ventilation becomes an urgent concern in a child with ARM or other emergency neonatal surgical conditions, as it is required for surgery. This challenging situation is further compounded in resource-poor settings, where appropriate equipment for intubation and ventilation maneuvers is unavailable. The initial mask ventilation and pre-oxygenation may be difficult due to a small mandible, difficulty in obtaining a mask seal, and the presence of a large tongue. This improves with the presence of an oropharyngeal airway. The space between the mask and mandible can be packed with gauze while performing a jaw lift to reduce air leaks. These maneuvers were largely successful in the index case, improving saturation to 95%-98%. Following this, difficulties in visualizing the vocal cords and lifting the epiglottis with the laryngoscope blade may be encountered. For these, some techniques have been described, including intubation through a laryngeal mask airway (LMA). This presents difficulties in removing the LMA (laryngeal mask airway) over the ETT. The use of video laryngoscopes, fiber-optic bronchoscopes, retrograde intubation, and tracheostomy has been described. However, due to limited hospital resources, the former may not be available. The latter (tracheostomy) is often reserved as a last option due to its invasiveness and higher complication rate, including bleeding, infection, erosion into vessels, vocal cord dysfunction, airway stenosis, and difficult decannulation (28%-51% success in children under one year at the time of cannulation), with an average of two years between cannulation and successful decannulation [10-11].

Adequately securing the airway in neonatal intestinal obstruction surgery also helps reduce the risk of aspiration. Due to the need for urgent surgical intervention in a setting without a well-equipped referral center, a high-risk approach was chosen after careful deliberation and parental counseling. Despite the success of this approach in the index patient, we wish to emphasize that this is in no way a recommendation. We also highlight the need for more concerted efforts to equip referral facilities with difficult airway sets, particularly for the pediatric age group, as pediatric surgical patients with all forms of difficult airway often present with associated conditions that constitute a surgical emergency and necessitate urgent intervention.

## Conclusions

This case represents a rare instance of anorectal malformation in a child with TCS. The need for urgent surgical intervention, combined with repeated failed intubations and the lack of facilities for managing difficult pediatric airways in a resource-poor setting, necessitated the unconventional use of a facemask to deliver anesthetic gas; this is in no way a recommended approach. There is a need for adequate preparedness, including the provision of difficult airway trays for the pediatric age group in cases such as this. The possible teratogenic effects of antiretroviral drugs also need to be studied further.
